# Effects of molecular structural variants on serum Krebs von den Lungen-6 levels in sarcoidosis

**DOI:** 10.1186/1479-5876-10-111

**Published:** 2012-07-11

**Authors:** Masahiko Shigemura, Yasuyuki Nasuhara, Satoshi Konno, Chikara Shimizu, Kazuhiko Matsuno, Etsuro Yamguchi, Masaharu Nishimura

**Affiliations:** 1 First Department of Medicine, Hokkaido University School of Medicine, Sapporo, Japan; 2 Division of Laboratory and Transfusion Medicine, Hokkaido University Hospital, Sapporo, Japan; 3 Division of Respiratory Medicine and Allergology, Aichi Medical University, Aichi, Japan; 4 First Department of Medicine, Hokkaido University School of Medicine, North 15, West 7, Kita-ku, Sapporo, 060-8638, Japan

**Keywords:** Serum KL-6, Molecular structural variant, Sarcoidosis

## Abstract

**Background:**

Serum Krebs von den Lungen-6 (KL-6), which is classified as human mucin-1 (MUC1), is used as a marker of sarcoidosis and other interstitial lung diseases. However, there remain some limitations due to a lack of information on the factors contributing to increased levels of serum KL-6. This study was designed to investigate the factors contributing to increased levels of serum KL-6 by molecular analysis.

**Methods:**

Western blot analysis using anti-KL-6 antibody was performed simultaneously on the bronchoalveolar lavage fluid (BALF) and serum obtained from 128 subjects with sarcoidosis.

**Results:**

KL-6/MUC1 in BALF showed three bands and five band patterns. These band patterns were associated with the *MUC1* genotype and the KL-6 levels. KL-6/MUC1 band patterns in serum were dependent on molecular size class in BALF. Significantly increased levels of serum KL-6, serum/BALF KL-6 ratio and serum soluble interleukin 2 receptor were observed in the subjects with influx of high molecular size KL-6/MUC1 from the alveoli to blood circulation. The multivariate linear regression analysis involving potentially relevant variables such as age, gender, smoking status, lung parenchymal involvement based on radiographical stage and molecular size of KL-6/MUC1 in serum showed that the molecular size of KL-6/MUC1 in serum was significant independent determinant of serum KL-6 levels.

**Conclusions:**

The molecular structural variants of KL-6/MUC1 and its leakage behavior affect serum levels of KL-6 in sarcoidosis. This information may assist in the interpretation of serum KL-6 levels in sarcoidosis.

## Background

**Figure 1:**
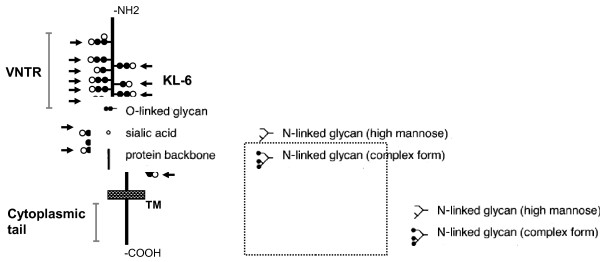
**Possible diagrammatic structure of MUC1 showing KL-6 epitopes.** TM, transmembrane; VNTR, variable number of tandem repeats. Arrows indicate KL-6 antigens on MUC1 protein.

Krebs von den Lungen-6 (KL-6) is a mucinous sialylated sugar chain on human mucin-1 (MUC1) [1,2]. MUC1 consists of a large extracellular domain, a single-pass transmembrane region, and an intracellular cytoplasmic tail [3,4]. The large extracellular domain contains a variable number of tandem repeat (VNTR) regions that are heavily glycosylated (Figure 1). In normal lung tissue, KL-6 is expressed on type II pneumocytes [1,5]. KL-6 is present in high concentrations in bronchoalveolar lavage fluid (BALF) and also circulates in blood [6]. Serum KL-6 is specifically elevated in a majority of patients with interstitial lung diseases (ILDs), and this phenomenon is considered to reflect the production by regenerating type II epithelial cells based on disease activity [6-13]. Therefore, measurement of serum KL-6 is widely accepted, particularly in Japan, as a diagnostic test for ILDs and a marker of disease activity.

Sarcoidosis is a multiorgan inflammatory disease of unknown origin that is characterized by noncaseating epithelioid cell granuloma and lymphocytic alveolitis [14]. Because the natural history and prognosis of sarcoidosis are unpredictable, it is important to monitor disease development during management [14]. KL-6 is considered to be one of the useful serum markers for monitoring diseases activity in sarcoidsis. Several investigators have reported that levels of serum KL-6 reflect lymphocytic alveolitis and increased parenchymal infiltration [11-13]. However, we have experienced some limitations in the interpretation of serum KL-6 levels, which include its dissociation with disease activity and different behavior from other serum markers in some cases. This prompted us to examine the factors contributing to increased levels of serum KL-6.

There are known variations in the length and structure of the MUC1 protein that result from polymorphisms in the VNTR [15,16]. The size class of MUC1 protein in tears is reported to be linked with the genotype of a single nucleotide polymorphism (SNP) in exon 2 (rs4072037) of the *MUC1* gene [16]. In addition, Janssen et al. recently reported an association between this polymorphism and variations in serum KL-6 levels in healthy individuals and patients with pulmonary sarcoidosis [17]. Based on these reports, we hypothesized that: 1) various molecular sizes of KL-6/MUC1, which are genetically determined by *MUC1* gene polymorphism (such as rs4072037), would be present in BALF; 2) the influx of KL-6/MUC1 from alveoli to blood is dependent on the molecular size of KL-6/MUC1; and finally, 3) serum KL-6 levels would be affected by the molecular size and leakage behavior of KL-6/MUC1, in addition to increased local production of KL-6/MUC1 in lung.

In this study, we examined the factors contributing to the variable increases in serum levels of KL-6 using molecular analysis in patients with sarcoidosis, all of whom simultaneously underwent blood sampling and bronchoalveolar lavage (BAL).

## Materials and methods

### Subjects

A total of 128 subjects with pulmonary sarcoidosis visiting the pulmonary clinic of the First Department of Medicine, Hokkaido University Hospital between April 2000 and July 2011 were enrolled into this study. The diagnosis of pulmonary sarcoidosis was established based on clinical findings and histologic evidence of noncaseating epithelioid cell granulomas, after excluding known causes of granulomatous diseases, in accordance with the American Thoracic Society/European Respiratory Society/World Association of Sarcoidosis and other Granulomatous Disorders guidelines [14]. All subjects underwent BAL, which is a routine diagnostic procedure at our hospital for patients with undiagnosed sarcoidosis, as described previously [18,19]. Serum samples were collected 30 minutes before BAL in all subjects. The study population, sex, age, smoking history, radiographical stage [14], BALF findings, pulmonary functions results, and levels of serum markers are shown in Table 1. The smoking status of one subject was unknown. BALF cell analysis of 1 patient was not performed due to problems with BAL storage. Pulmonary function data were available from 122 patients.

**Table 1:** Characteristics of the study population

	Sarcoidosis
No. of subjects	128
Men/Women	34/94
Age, yr	55(17–79)
Cigarette smoking	55/29/43
(never/former/current)	
Radiograhical stage	21/56/43/8
(0/I/II/III)	
BALF findings	
Total cell counts, 10^4^/mL	18.1 (3.2–92.2)
Macrophages, %	66.3 (6.0–97.0)
Lymphocytes, %	32.8 (2.9–76.3)
CD4/CD8, ratio	4.93 (0.65–30.16)
Pulmonary function result	
VC, % predicted	113.0 (70.5–153.7)
D_Lco_, % predicted	90.1 (37.0–142.0)
Serum maker	
soluble IL-2 receptor, U/mL	805 (117–4990)
KL-6 levels	
BALF, U/mL	296 (90–1507)
serum, U/mL	336 (102–3091)

Data are presented as median (range).

All patients had provided written informed consent for their samples to be used in future clinical research [18,19]. The Institutional Review Board of Hokkaido University Hospital for Clinical Research approved the study protocols (approval No. 009–0295).

### Western Blotting

Western blotting was performed on BALF and serum from all subjects. Briefly, protein samples from BALF and serum were electrophoresed on 3%–8% NUPAGE Tris-acetate gels (Invitrogen, Carlsbad, CA) and were transferred to nitrocellulose membranes (Invitrogen). Membranes were blocked with PBS containing 3% skim milk. Western blot analysis was performed using anti-KL-6 antibody (anti-KL-6 antibody was kindly provided by Sanko Junyaku Co., Ltd.) and DF-3 antibody (Santa Cruz Biotechnology, Santa Cruz, CA) followed by alkaline phosphatase-conjugated goat anti-mouse Ig. Bands were developed using the WesternBreeze Chromogenic Immunodetection Kit (Invitrogen).

### Genotyping of *MUC1* polymorphism

The *MUC1* SNP (exon 2; rs4072037) was genotyped in 80 patients with sarcoidosis, who had consented to future genetic studies, using the TaqMan system (Assay ID: C_27532642_10, Applied Biosystems, Foster City, CA).

### Measurement of KL-6, albumin and soluble interleukin 2 receptor

KL-6 levels in both BALF and serum were measured by electrochemiluminescent immunoassay using the PICOLUMI KL-6 kit (Sanko Junyaku, Tokyo, Japan). Reference intervals were 105.3–401.2 U/mL for Japanese normal subjects. Albumin levels in BALF were measured on a Hitachi 7070 automated analyzer with TAC-2 test Albumin U (Medical and biological laboratories co., LTD., Nagoya, Japan). Serum soluble interleukin 2 (IL-2) receptor was measured by a solid-phase, two-site chemiluminescent immunometric assay (IMMULITE 2000 IL2R, Siemens Healthcare Diagnostics, Los Angeles, CA).

### Statistical methods

Statistical analysis was performed with SYSTAT 11 for Windows (Systat Inc., Chicago, IL) and SAS (SAS Institute, Inc., Cary, NC). Data were expressed as median and ranges. All data were not normally distributed on univariate analysis, the natural logarithm of all data were used for further statistical analyses. Comparisons were performed by unpaired *t*-test or ANOVA adjusting potentially relevant variable such as cigarette smoking when assessing the influence of smoking status (never, ex or current). Differences between groups were evaluated by ANOVA and were assessed by Bonferroni post-hoc test. The relationship between leakage behavior of KL-6/MUC1 and smoking status was assessed using *χ*^2^-test. Correlations between different parameters were determined by Pearson’s correlation coefficient. We used Haploview software version 4.1 (http://www.broad.mit.edu/mpg/haploview; Barrett et al. 2005) in order to compare the observed numbers of genotypes with the number of expected genotypes under Hardy-Weinberg equilibrium using *χ*^2^-test. The association between *MUC1* genotypes and KL-6/MUC1 band patterns in BALF was assessed using Cochran-Mantel-Haenszel Statistics. In order to identify independent factors predictive for the serum KL-6 levels, the multivariate linear regression analysis involving potentially relevant variables was performed. A *p* value of <0.05 was regarded as significant.

## Results

### Western blot analysis

**Figure 2:**
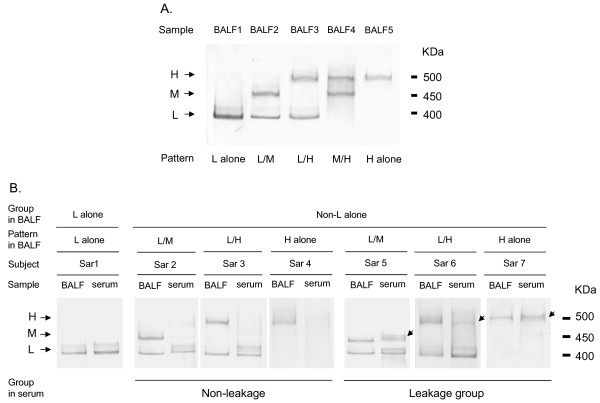
**A. Representative examples of Western blot analyses using anti-KL-6 antibody in BALF.** Three bands were detected under reducing conditions with low (L), middle (M) and high molecular weights (H), corresponding to approximately 400, 450 and 500 kDa, respectively. Based on the three bands, there were five band patterns (L alone, L/M, L/H, M/H, H alone) identified in 128 subjects with sarcoidosis. **B. Comparison of KL-6/MUC1 band patterns between BALF and serum in 7 representative subjects.** Sar, subjects with sarcoidosis. Arrows indicate M or H bands present in serum.

**Figure 3:**
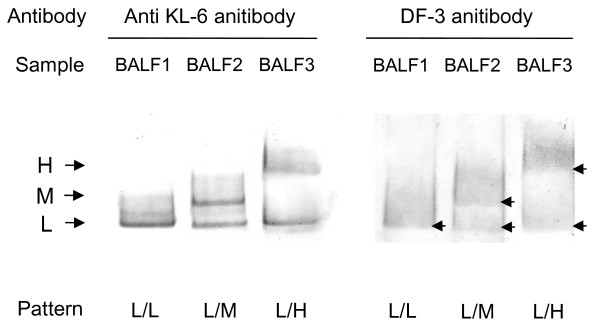
**Representative examples of Western blot analysis using anti-KL-6 antibody and DF-3 antibody in BALF.** A, anti-KL-6 antibody; B, DF-3 antibody. Arrows indicate L, M or H bands.

Western blot analysis of BALF with anti-KL-6 antibody revealed three bands (low molecular size (L), middle molecular size (M) and high molecular size (H), at approximately 400, 450 and 500 kDa, respectively) under reducing conditions. Furthermore, based on the combination of these bands, five band patterns (L alone, L/M, L/H, M/H and H alone) were identified (Figure 2A). In addition, Western blot analysis with DF-3 antibody showed the same band patterns corresponding to the KL-6/MUC1 band patterns in BALF (Figure 3). All Subjects who displayed the L alone pattern in BALF also displayed L alone bands in serum (Figure 2B; Sar 1). On the other hand, subjects who displayed non-L alone patterns in BALF showed diverse patterns in serum. Based on the similarities and differences in band patterns between BALF and serum, we classified these subjects into two groups; “non-leakage” and “leakage”. The non-leakage group displayed only L bands or neither M nor H bands in serum, despite the presence of M or H bands in BALF (Figure 2B; Sar 2, 3 and 4). In contrast, the leakage group displayed identical band patterns between BALF and serum (Figure 2B; Sar 5, 6 and 7). The frequency and percentage of KL-6/MUC1 band patterns in BALF and in serum are summarized in Table 2. Thirty-one of 47 subjects with non-L alone in BALF (66.0%), including 5 subjects in whom bands were not detected in serum, were classified as “non-leakage”. The leakage behavior of high molecular size KL-6/MUC1 (i.e., M or H band) appeared to be influenced by smoking status (never, ex or current), but it did not reach statistical significance (*χ*^2^-test, *p* > 0.05) (Table 3).

**Table 2:** The frequency and percentage of KL-6/MUC1 band patterns in between BALF and serum

		Band pattern in serum						
			L alone	L/M	L/H	H alone	Not detected	Total
Band pattern in BALF	L alone		81					81
			100.0%					100.0%
	Non L alone	L/M	8 *	8 **				16
			50.0%	50.0%				100.0%
		L/H	18 *		6 **			24
			75.0%		25.0%			100.0%
		M/H					3 *	3
							100.0%	100.0%
		H alone				2 **	2 *	4
						50.0%	50.0%	100.0%
Total			107	8	6	2	5	128
			83.6%	6.3%	4.7%	1.6%	3.9%	100.0%

*, non-leakage group; **, leakage group.

**Table 3:** Relationship between leakage behavior of high molecular size KL-6/MUC1 and smoking status

		Leakage behavior of high molecular size KL-6/MUC1		Total
		Non-leakage	Leakage	
Smoking status	Non/Ex smoker	22	8	30
		73.3%	26.7%	100.0%
	Current smoker	9	8	17
		52.9%	47.1%	100.0%
Total		31	16	47
		66.0%	34.0%	100.0%

*X*^2^-test, *p* > 0.05.

### Relationship between KL-6 levels and KL-6/MUC1 size class

**Figure 4:**
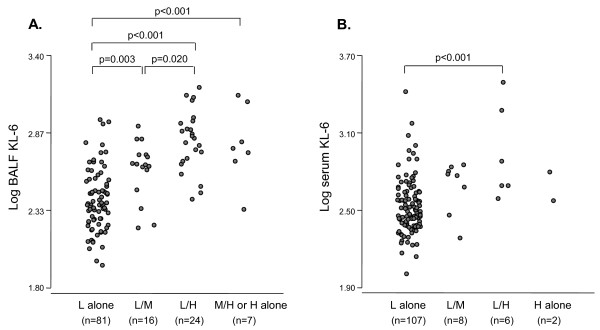
A. Comparison of KL-6 levels in BALF based on KL-6/MUC1 band pattern in BALF. B. Comparison of KL-6 levels in serum based on KL-6/MUC1 band pattern in serum.

BALF KL-6 levels in subjects with the H alone or M/H, L/H and L/M patterns in BALF were significantly higher than those with the L alone pattern in BALF (*p* < 0.001, <0.001 and 0.003, respectively) (Figure 4A), thus suggesting that larger molecular size of KL-6/MUC1 is associated with higher levels of BALF KL-6. Similarly, serum KL-6 levels in subjects with the L/H pattern in serum were significantly higher than those with the L alone pattern (*p* < 0.001) (Figure 4B).

### Relationship between *MUC1* genotypes and KL-6/MUC1 size

We examined the allele frequency of rs4072037. There was 129 (80.6%) for A and 31 (19.4%) for G. The genotype frequencies were 53 (66.3%) for AA, 23 (28.7%) for AG, and 4 (5.0%) for GG. No significant deviation from the Hardy-Weinberg equilibrium was observed (*p* > 0.05). When the KL-6 levels in both serum and BALF were grouped according to the genotype, the results were AA (serum: 285U/mL, 102–2627; BALF: 240U/mL, 90–1224), AG (serum: 480U/mL, 183–3091, BALF: 662U/mL, 130–1336) and GG (serum: 437U/mL, 337–1600; BALF: 444U/mL, 219–633) The KL-6 levels in both serum and BALF from the subjects with AG genotype were higher than those with AA genotype (*p* < 0.001, <0.001, respectively). Table 4 shows the relationship between *MUC1* genotypes and KL-6/MUC1 band patterns in BALF. There is a significant relationship between genotype and KL-6/MUC1 band patterns in BALF (Cochran-Mantel-Haenszel Statistics, *p* < 0.001); the A allele is linked with L bands and the G allele with H bands.

**Table 4:** Relationship between MUC 1 gene polymorphism (rs4072037) and KL-6/MUC 1 band patterns in BALF

		KL-6/MUC1 band pattern in BALF					
		L alone	L/M	L/H	M/H	H alone	Total
rs4072037	AA	47	5	1			53
		88.7%	9.4%	1.9%			100.0%
	AG	1	6	15	1		23
		4.3%	26.1%	65.2%	4.3%		100.0%
	GG		1		1	2	4
			25.0%		25.0%	50.0%	100.0%
Total		48	11	17	2	2	80
		60.0%	13.8	21.3%	2.5%	2.5%	100.0%

Cochran-Mantel-Haenszel Statistics, *p* < 0.001.

### Determination of leakage behavior of high molecular size KL-6/MUC1

**Figure 5:**
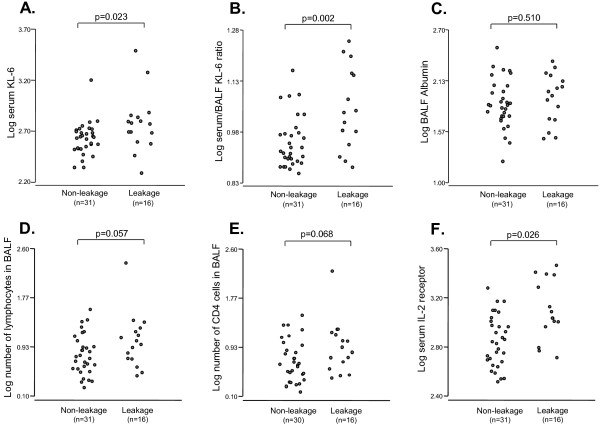
**A, B, C, D, E and F. Comparison of serum KL-6 levels, serum/BALF KL-6 ratio, BALF albumin levels, number of lymphocytes and CD**_**4**_**positive cells in BALF and serum soluble IL-2 receptor levels between non-leakage and leakage in subjects who displayed non-L alone band pattern in BALF.**

As the subjects who displayed non-L alone patterns in BALF showed diverse band patterns in serum, we examined the presence of differences in the leakage behavior of high molecular size KL-6/MUC1 (i.e., M or H band). We compared the serum KL-6 levels, the serum/BALF KL-6 ratio, the BALF albumin levels, the numbers of lymphocytes and CD4_4_ positive cells in BALF and the serum soluble IL-2 receptor levels between the non-leakage and the leakage groups. As expected, the serum KL-6 levels in the leakage group were significantly higher than those in the non-leakage group (*p* = 0.023, Figure 5A). In addition, there was a significant increase in the serum/BALF KL-6 ratio in the leakage group, when compared with the non-leakage group (*p* = 0.002, Figure 5B). This result remained significant even after controlling for the smoking status (ANOVA, *p* = 0.005). In contrast, no significant differences in the BALF albumin levels between the non-leakage and the leakage groups were observed (*p* = 0.510, Figure 5C). The leakage group tended to have the slightly higher numbers of lymphocytes and CD_4_ positive cells in BALF than the non-leakage group (*p* = 0.057 and 0.068, respectively) (Figure 5D, E). In addition, the serum soluble IL-2 receptor levels in the leakage group was significantly higher than the non-leakage group (*p* = 0.026, Figure 5F).

### Impact of KL-6/MUC1 molecular structural variants on serum KL-6 levels

Recent reports have shown that serum KL-6 levels may be affected by several potential factors, such as age, gender and smoking status, other than lung diseases [17,20]. In addition, previous reports have found that radiographical staging showing parenchymal involvement (stages II and higher) is correlated with significantly higher KL-6 levels, as compared to patients without parenchymal involvement (stages 0 and I) [10-12]. In univariate analyses between serum KL-6 levels and potentially relevant variables, there were significant differences between the subjects without and with lung parenchymal involvement based on radiographical stage [14] (*p* = 0.020) and those with low and higher molecular size KL-6/MUC1 in serum (*p* < 0.001) (Table 5). In order to identify independent factors predictive for the serum KL-6 levels, the multivariate linear regression analysis involving potentially relevant variables such as age (years), gender (male or female), smoking status (never, ex or current), lung parenchymal involvement (without or with parenchymal infiltration) and molecular size of KL-6/MUC1 in serum (low or higher molecular size) was performed. The multivariate linear regression analysis showed that only molecular size of KL-6/MUC1 in serum (Standardized β coefficient = 0.365, *p* < 0.001) was significant independent determinant of serum KL-6 levels (Table 6).

**Table 5:** Univariate analyses between Log serum KL-6 and potentially relevant variables

		Correlation with Log serum KL-6	
		R*	*P*-value
Age		0.065	0.466
			
		Mean ± SD	*P*-value**
Gender	Male	2.57 ± 0.237	
			0.726
	Female	2.56 ± 0.264	
			
Smoking status	Non/Ex smoker	2.54 ± 0.205	
			0.072
	Current smoker	2.62 ± 0.302	
			
Lung parenchymal involvement	Without	2.53 ± 0.198	
			0.020
	With	2.63 ± 0.291	
			
Band pattern in serum	Low molecular size	2.54 ± 0.223	
			<0.001
	Higher molecular size	2.78 ± 0.284	

**Table 6:** Independent factors predictive for serum KL-6 levels

	Standardized β coefficient	*P*-value
Age	0.040	0.673
Gender	−0.025	0.782
Smoking status	0.087	0.353
Lung Parenchymal involvement*	0.125	0.164
Molecular size of KL-6/MUC1 in serum	0.365	<0.001

* Based on radiographical stage.

## Discussion

The increased levels of serum KL-6 in patients with ILDs are thought to result from an increased local production of KL-6/MUC1 in lung and enhanced permeability following the destruction of the alveolar-blood interface [5,21]. In this study, we discovered three important findings with regard to the factors contributing to the variably in serum levels of KL-6. First, KL-6/MUC1 in BALF showed three bands and five band patterns, and these band patterns were associated with *MUC1* genotype and KL-6 levels. Second, the KL-6/MUC1 band patterns in serum were dependent on molecular size class in BALF, as proteins of different size classes did not pass through the alveolar-blood interface in a similar manner. Finally, the molecular structural variants of KL-6/MUC1 and its leakage behavior affect serum KL-6 levels. We believe that what we found in this study would provide new insight into understanding the limitations related to the measurement of serum KL-6.

Before further discussion on these issues, we should comment on the limitations of this study. It is possible that the size classes of KL-6/MUC1 detected by Western blot analysis using anti-KL-6 antibody were affected by glycosylation of MUC1, and may not strictly reflect molecular weight. However, Western blot analysis using DF-3 antibody, which recognize the core peptide of MUC1 [22,23], displayed the same band patterns, suggesting that the glycosylation of MUC1 had little influence on the molecular size of KL-6/MUC1 characterized by Western blot analysis using anti-KL-6 antibody. We could not examine the local production of KL-6/MUC1 and quantitatively analyze each molecular size of KL-6/MUC1 in this study. The local production of KL-6/MUC1 may still be important for the interpretation of serum KL-6 levels.

The larger MUC1 proteins may express more KL-6 on its surface than the smaller MUC1 proteins. In this study, KL-6 levels in BALF, which are little influenced with alveolar-blood interface, from the subjects with larger molecular size of KL-6/MUC1 (i.e. M and H band) were significantly higher than those with low molecular size (i.e. L band). Janssen et al. reported that the *MUC1* genotype was of strong influence on serum KL-6 levels in the Caucasian population and also speculated that the positive association between *MUC1* gene polymorphism and serum KL-6 levels is caused by *MUC1* allele-related molecular size. The *MUC1* genotype was clearly linked with KL-6/MUC1 band pattern and related to KL-6 levels in our study. Therefore, KL-6 levels are affected by genetically determined molecular sizes of KL-6/MUC1. Furthermore, our results showed that significantly increased levels of serum KL-6 were observed in the subjects with influx of high molecular size KL-6/MUC1 from the alveoli to blood circulation. This is the first reports describing the influence of leakage behavior of high molecular size KL-6/MUC1 on serum KL-6 levels. Considering for the molecular size of KL-6/MUC1 and its leakage behavior may increase the value of serum KL-6 as a marker of sarcoidosis.

With regard to *MUC1* gene polymorphism and KL-6/MUC1 band patterns in BALF, although the A allele was linked with the L band and the G allele was linked with the H band, 5 of 53 subjects (9.4%) with the AA genotype, 7 of 23 subjects (30.4%) with the AG genotype and 1 of 4 subjects (25.0%) with the GG genotype exhibited the M band. *MUC1* alleles can mostly be divided into size classes containing small (30–45) or large (60–90) numbers of repeats on Southern blot analysis [15,24]. In other words, the molecular size of the protein, which directly reflects the allele length, is associated with the number of tandem repeats on VNTR regions. We thus speculate that the presence of M bands in BALF indicates the presence of intermediate numbers of tandem repeats in VNTR regions.

Influx mechanism of KL-6/MUC1 from the alveoli to blood circulation is unclear. In sarcoidosis, increased levels of BALF albumin are thought to result from an influx of plasma albumin into the alveoli [25]. To analyze the leakage behavior of high molecular size KL-6/MUC1, we also measured the concentration of BALF albumin in subjects with non-L alone pattern in BALF. No significant differences between the groups of non-leakage and leakage of high molecular size KL-6/MUC1 were observed in the BALF albumin levels. In contrast, there was a significant increase in the serum/BALF KL-6 ratio, which is an indicator of leakage behavior, in the leakage group. The influx of the high molecular size KL-6/MUC1 may be not parallel to albumin-size proteins and be complicated.

The passage of high molecular size KL-6/MUC1 from alveoli to blood may be regulated by pore size and/or electrostatic forces [26-29]. In an isolated dog lung, Conhaim et al. proposed that the lung epithelial barrier is best described by a three-pore-size model, including a very small number of large pores (400-nm radius), an intermediate number of medium-size pores (40-nm radius), and a very large number of small pores (1.3-nm radius) [28]. If such a theory could be extrapolated to the human lung, it is possible that high molecular size KL-6/MUC1 passes through the alveolar-blood interface, considering that the molecular diameter of KL-6/MUC1 is approximately 200–500 nm [4]. A number of studies have indicated that the surfaces of endothelium, epithelium and basement membranes are covered by negatively charged proteoglycans. Under such conditions, electrostatic forces would affect the movement of charged versus uncharged macromolecules [29,30]. As most lung proteins, particularly mucins, are negatively charged at physiologic pH [31,32], it is very plausible that similar electrostatic repulsion influences their transfer from lung into blood. Hence, we speculate that electrostatic forces would limit the transfer of high molecular size KL-6/MUC1 more efficiently at the alveolar-blood interface, as compared to small molecular size, under healthy conditions. However, if such barrier function is damaged, the high molecular size proteins would more easily pass through the alveolar blood interface. In our study, the numbers of lymphocytes in BALF tended to be elevated and the serum IL-2 receptor levels were significantly increased in the subjects in which the influx of the high molecular size KL-6/MUC1 was observed. The percentage of lymphocytes in BALF is thought to be a marker of alveolitis [33,34]. Soluble IL-2 receptor was reported to be associated with T-lymphocyte alveolitis [35] . Our results suggest that high molecular size KL-6/MUC1 might transfer from the alveoli to blood circulation as a result of alveolitis.

Cigarette smoke exposure is known to increase the permeability of the lung epithelial/endothelial barrier [36,37]. The mechanisms by which cigarette smoke disrupts epithelial integrity have not been fully defined, but are likely to involve alterations in the function of the tight junctions [38], which normally maintain the polarity of the epithelial cells and limit flow of ions and proteins from one side of the monolayer to the other. In this study, we did not demonstrate statistically significant relationship between smoking status and the leakage behavior of high molecular size KL-6/MUC1. The multivariate linear regression analysis showed that the molecular size of KL-6/MUC1 in serum was only significantly independent determinant of serum KL-6 levels. However, there have been a few reports describing that serum KL-6 levels may be affected by smoking status in healthy controls [17,20]. Further studies to analyze relationships between cigarette smoking and leakage behavior of KL-6/MUC1 and KL-6 levels in healthy controls would be necessary.

The findings of this study are important for both the interpretation of serum KL-6 levels and the consideration of serum marker proteins, such as surfactant protein (SP)-D [39], that originate in lung epithelium and have been identified in serum from patients with sarcoidosis and other ILDs. Leth-Larsen et al. reported that SP-D gene polymorphism influences the potential for oligomerization, which results in significantly different SP-D serum levels [40]. Their data strongly indicates that the passage of SP-D protein through the alveolar-blood interface is also dependent on the size of the SP-D protein corresponding to the gene polymorphism. With regard to serum markers in lung diseases, the different behaviors among markers have long been a subject of debate. The molecular size classes that may be specific to each marker protein could explain the different behaviors of these proteins in serum.

Finally, it is noteworthy that the minor allele (i.e., G allele) frequency of rs4072037 may differ with ethnicity. In the Caucasian population, Janssen et al. reported that it was 0.45 [17], which is much higher than our observed frequency of 0.21, and the frequency in the Japanese population reported by HapMap (http://www.hapmap.org). Thus, the interpretation of serum KL-6 levels may be more complex in the Caucasian population than in Japanese population. This may explain the fact that measurements of KL-6 are not well accepted as diagnostic markers of sarcoidosis and other interstitial lung diseases in most Western countries, in contrast to Japan, where such measurements are routinely used in various clinical settings.

## Conclusions

This study has shown that the molecular structural variants of KL-6/MUC1 and its leakage behavior affect changes in serum levels of KL-6 in sarcoidosis. This information will assist in the interpretation of serum KL-6 levels in sarcoidosis. Further studies to examine the factors contributing to the variable increases in serum levels of KL-6 using molecular analysis in other ILDs would be warranted.

## Abbreviations

BAL: Bronchoalveolar lavage; BALF: Bronchoalveolar lavage fluid; DLco: Diffusing capacity of lung for carbon monoxide; ILDs: Interstitial lung diseases; KL-6: Krebs von den Lungen-6; MUC1: Mucin-1; SNP: Single nucleotide polymorphism; SP-D: Surfactant protein-D; VA: Alveolar volume; VC: Vital capacity; VNTR: Variable number of tandem repeat.

## Competing interests

The authors declare that they have no competing interests.

## Authors’ contributions

MS, YN, SK, MN contributed to the study design, data analysis, data collection, data interpretation, figures and writing of the manuscript. CS and KM contributed to the study design, data interpretation, and writing of the manuscript. EY contributed to sample collection and interpretation of the data and writing of the manuscript. All authors have read and approved the final manuscript.
